# Crystal engineering of analogous and homologous organic compounds: hydrogen bonding patterns in trimethoprim hydrogen phthalate and trimethoprim hydrogen adipate

**DOI:** 10.1186/1860-5397-2-8

**Published:** 2006-04-07

**Authors:** Packianathan Thomas Muthiah, Savarimuthu Francis, Urszula Rychlewska, Beata Warżajtis

**Affiliations:** 1 Department of Chemistry, Bharathidasan University, Tiruchirappalli-620 024, India; 2 Department of Chemistry, Adam Mickiewicz University, Grunwaldzka 6, 60–780 Poznañ, Poland

## Abstract

**Background:**

Trimethoprim [2,4-diamino-5-(3',4',5'-trimethoxybenzyl)pyrimidine] is an antifolate drug. It selectively inhibits the bacterial dihydrofolate reductase (DHFR) enzyme.

**Results:**

In the crystal structures of trimethoprim (TMP)-hydrogen phthalate (**1**) and trimethoprim-hydrogen adipate (**2**), one of the N atoms of the pyrimidine ring is protonated and it interacts with the deprotonated carboxylate oxygens through a pair of nearly parallel N-H...O hydrogen bonds to form a fork-like interaction. In the compound **1**, the pyrimidine moieties of the TMP cations are centrosymmetrically paired through a pair of N-H...N hydrogen bonds involving 4-amino group and the N (N3) atom of the pyrimidine rings to form a 8-membered hydrogen bonded ring [R^2^_2_(8)]. The 4-amino group of one TMP moiety and 2-amino group of another TMP moiety (both moieties are members of a base pair) are bridged by the carbonyl oxygen of the phthalate moiety through N-H...O hydrogen bonds forming 8-membered hydrogen-bonded ring [R^2^_2_(8)]. The characteristic hydrogen-bonded rings observed in the structure aggregate into a supramolecular ladder consisting of a pair of chains, each of which is built up of alternate TMP and hydrogen phthalate ions. In the compound **2**, two TMP cations and two hydrogen adipate anions are arranged about an inversion center so that the complementary DDAA (D = donor, A = acceptor) arrays of quadruple hydrogen-bonding patterns are formed. The head-to-tail arrangement of the hydrogen adipate ions leads to a hydrogen-bonded supramolecular chain. From crystal engineering point of view, it is interesting to note that the compound **1** has a hydrogen-bonded network remarkably identical with its aliphatic analogue, trimethoprim hydrogen maleate. Similarly the compound **2**, resembles its homolog trimethoprim hydrogen glutarate.

**Conclusion:**

In the crystal structure of trimethoprim hydrogen phthalate, the hydrogen-bonded network is remarkably identical with its aliphatic analogue, trimethoprim hydrogen maleate. Similarly in the crystal structure of trimethoprim hydrogen adipate the hydrogen bonded network resembles its homolog trimethoprim hydrogen glutarate.

## Introduction

Non-covalent interactions are the essential tool for both crystal engineering and supramolecular chemistry [1–4]. Supramolecular synthons are the building motif for these fields [5]. Hydrogen bonding is the most important non-covalent interactions. It plays a vital role in biological structure and functions, molecular design, etc [6]. Recently Sijbesma and Meijer have investigated the role of quadruple hydrogen bonded network in the various heterocyclic compounds [7]. Pyrimidine derivatives offer multiple metal binding modes and have remarkable hydrogen bonding potential. Trimethoprim [2,4-diamino-5-(3',4',5'-trimethoxybenzyl)pyrimidine] (TMP) is an antifolate drug. In the protonated form, it exerts its activity through the inhibition of the enzyme dihydrofolate reductase (DHFR) [8]. In most of the trimethoprim-carboxylate salts, one of the nitrogen atoms of the pyrimidine ring is protonated and it interacts with the carboxylate group through a nearly parallel N-H...O hydrogen bonds to form a cyclic bimolecular hydrogen bonded motif(fork-like interaction) [9–15]. These motifs self assemble in combination with other hydrogen-bonding groups leading to base-pairing, quadruple hydrogen bonded arrays, DDAA and DADA (D- donor, A = acceptor), etc. C-H...π, π-π stacking, etc are further stabilizing the crystal structures. The quadruple hydrogen bonded arrays have been observed in the crystal structures of TMP m-chlorobenzoate, [11] TMP-sorbate dihydrate [12], TMP-trifluoroacetate [15], TMP-formate [16], TMP-hydrogen glutarate [17], TMP-nitrate [18], TMP-salicylate methanol solvate [19] etc,. In the crystal structures of TMP-terephthalate-terephthalic acid [13], TMP-3-carboxy-4-hydroxybenzenesulfonate dihydrate [14] and TMP-sulfate trihydrate [20] other types of hydrogen-bonded networks are present.

The hydrogen-bonding networks in the crystal structures of TMP/pyrimethamine salts of various dicarboxylic acids have been investigated in our laboratory [13,21]. Recently we have also reported a novel isomorphism [21]. The crystal structures of pyrimethamine hydrogen maleate [21] and pyrimethamine hydrogen succinate [21] are isomorphous since the hydrogen succinate is the saturated analogue of hydrogen maleate. The hydrogen succinate ion adopts a folded conformation with an intramolecular hydrogen bond (mimicking the hydrogen maleate ion) leading to identical hydrogen-bonded networks in both the crystal structures. In the present work, crystal structures of TMP hydrogen phthalate and TMP hydrogen adipate have been investigated in order to identify the hydrogen bonding networks and compare them with those in the aliphatic analogue, TMP hydrogen maleate [22] and the homolog, TMP hydrogen glutarate [17] respectively.

## Results and discussion

**Figure 1:**
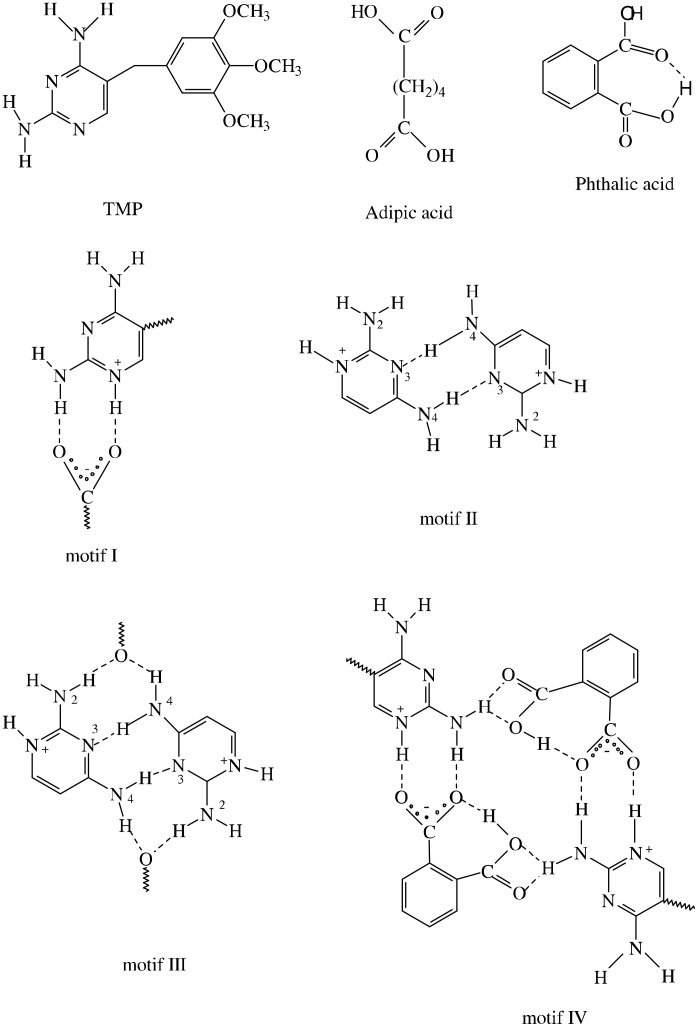
The schematic diagram for the various hydrogen-bonded motifs observed in compound (**1**).

**Figure 2:**
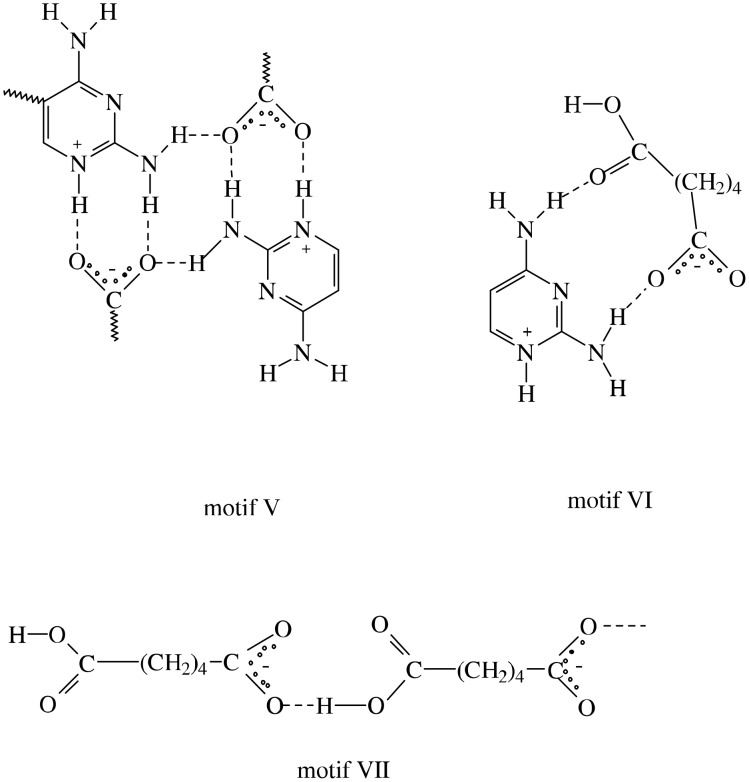
The schematic diagram for the various hydrogen-bonded motifs observed in compound (**2**).

**Figure 3:**
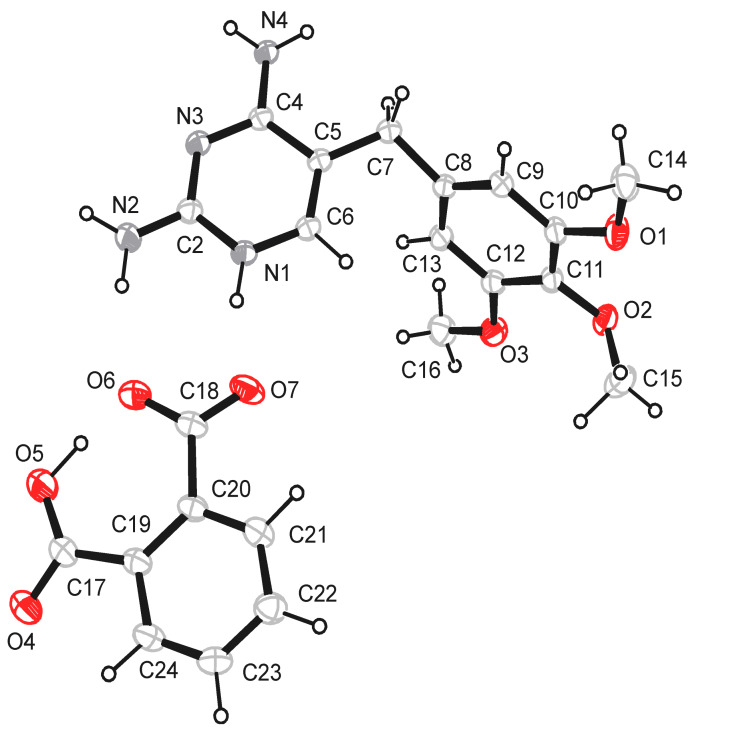
The ORTEP 3 view of the asymmetric unit of the compound **1**.

**Figure 4:**
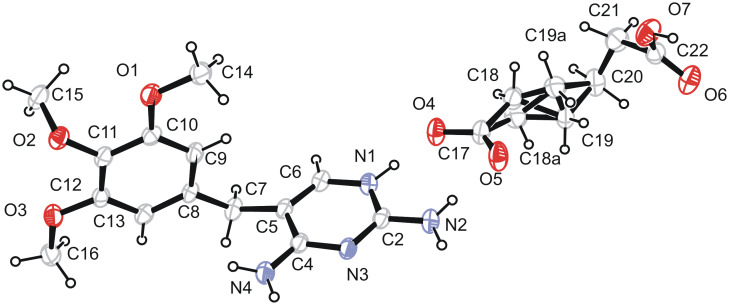
The ORTEP 3 view of the asymmetric unit of the compound **2**.

The schematic diagram of the hydrogen-bonded motifs observed in these crystal structures (see Supporting Information File 1) is shown in Figure 1 and Figure 2. An ORTEP 3 view of the compounds **1** & **2** is shown in Figure 3 and Figure 4. In the compounds **1** (trimethoprim hydrogen phthalate) and **2** (trimethoprim hydrogen adipate)(see Supporting Information File 2) one of the nitrogen atoms (N1) of the pyrimidine ring is protonated. The protonated pyrimidine ring interacts with the carboxylate oxygens through a pair of parallel N-H...O (Table 2) hydrogen bonds to form a fork-like interaction (motif I). This is reminiscent of the trimethoprim(TMP)-carboxylate interaction observed in the DHFR-TMP complexes [24]. This hydrogen bonded motif is one among the 24 most frequently observed bimolecular cyclic hydrogen-bonded motifs in organic crystal structures [25]. This has also been observed in the crystal structures of trimethoprim carboxylates such as trimethoprim salicylate monohydrate [26], trimethoprim acetate [9], trimethoprim salicylate methanol solvate [19], trimethoprim benzoate [10] etc,. This fork-like hydrogen-bonded interaction (motif I) has further self assembled in combination with other hydrogen-bonded motifs to form different types of networks. The planes of the carboxylate group and the pyrimidine ring (involved in the fork-like interaction) make a dihedral angle of 9.8° in compound **1** and 6.3° in compound **2** respectively.

**Table 1:** Crystallographic parameters for **1** and **2**

Properties	**1**	**2**

Formula	C_14_ H_19_ N_4_ O_3_^+^	C_14_ H_18_ N_4_ O_3_^+^
	C_8_ H_5_ O_4_^-^	C_6_ H_10_ O_4_^-^
M.wt	456.45	436.46
Crystal System	Triclinic	Triclinic
Space group	P-1	P-1
a/A°	4.6510(10)	8.172(2)
b/A°	11.700(2)	9.4744(8)
c/A°	20.362(4)	13.8162(8)
α/°	76.21(3)	85.741(6)
β/°	86.23(3)	87.430(10)
γ/°	84.03(3)	88.680(10)
V/A°^3^	1069.3(4)	1065.5(3)
Z	2	2
Radiation α/A°	1.54718	1.54718
Dc/g cm^-3^	1.418	1.360
T/K	293(2)	294(2)
μ/mm^-1^	0.900	0.870
F(000)	480	464
Reflection collected	4420	3706
Observed data [I>2σ(I)]	3137	3062
Parameters refined	395	404
Final R_1_ on observed data	0.0421	0.0439
Final wR_2_ on observed data	0.1246	0.1270
Structure solution	SHELXS97 [36]	SHELXS97
Structure refinement	SHELXL97	SHELXL97
Graphics	PLATON97 [37]	PLATON97

**Table 2:** Hydrogen bonding geometry for the compounds **1** and **2**

Compound	D-H...A	H...A	D...A	D-H...A

**1**	N4 - H3... O4^a^	2.19(2)	2.939(2)	139.8(17)
	N2 - H6... O6^b^	2.08(2)	2.954(2)	164(2)
	N4 - H9... N3^c^	2.11(2)	2.986(2)	171(2)
	N2 - H10... O4^d^	2.05(3)	3.008(2)	174(2)
	N2 - H10... O5	2.53(2)	3.109(2)	118.8(18)
	N1 - H11... O7	1.76(2)	2.678(2)	164.4(19)
	O5 - H16... O6	1.35(3)	2.399(2)	174(3)
	C21 - H5 ... O7	2.24(2)	2.670(2)	104.5(15)
	C24 - H8 ... O4	2.28(2)	2.679(3)	103.0(13)
	C15 - H25... O1	2.56(3)	3.037(3)	110(2)
**2**	N1 - H5O ...O4^e^	1.73(2)	2.682(2)	175(2)
	O7 - H7O... O4^f^	1.65(4)	2.555(2)	169(3)
	N2 - H21... O5	1.95(2)	2.841(2)	179(3)
	N2 - H22...O5	2.04(3)	2.833(2)	143(2)
	N4 - H42...O6	2.08(3)	2.951(3)	168(2)

Symmetry Codes : a = -1+x, 1+y, z, b = 1+x, y, z, c = -x, 1-y, 1-z, d = 1-x, -y, 1-z, e = 2-x, 1-y, -z, f = -1+x, y, z

**Figure 5:**
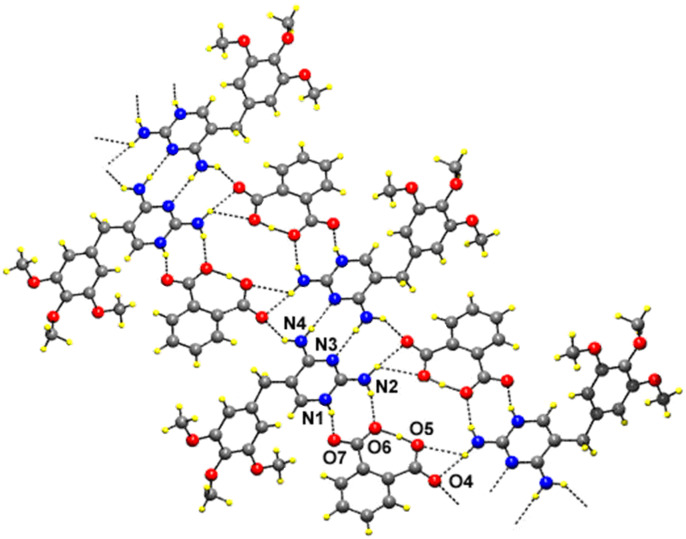
The hydrogen-bonded supramolecular ladder in the compound **1**.

In the compound **1** (Table 1), the pyrimidine moieties of trimethoprim cations are centrosymmetrically paired through a couple of N-H...N hydrogen bonds involving the 4-amino group and the N3 atom (motif II). One of the O atoms (O4) at the carboxyl group of the hydrogen phthalate ion bridges the 2-amino and 4-amino groups on either side of the paired TMP cations, forming 8-membered hydrogen-bonded ring (motif III), with graph-set notation [R^2^_2_(8)] [27]. The atom O5 of the carboxyl group of the hydrogen phthalate ion forms an intramolecular O-H...O hydrogen bond with the O6 atom of the carboxylate group. The hydrogen-bonding patterns formed upon the association of pyrimidine moieties of TMP molecules via self-pairing and carboxylate bridging resemble those observed in the crystal structure of TMP-hydrogen maleate [22]. The same type of DADA array has also been observed in the other crystal structures of trimethoprim-salicylate methanol solvate [18], trimethoprim-trifluoroacetate [15], pyrimethamine-hydrogen phthalate [21] etc,. The characteristic hydrogen-bonded rings observed in the structure aggregate into a supramolecular ladder consisting of a pair of chains, each of which is built up of alternate TMP and hydrogen phthalate ions (motif III & IV) as shown in Figure 5 [28]. The one of the hydrogen atoms of the 2-amino group is also involved in bifurcated hydrogen-bonding with the carboxyl O atoms (O4 & O5) to form a 4-membered hydrogen bonded ring [R^2^_1_(4)] [27].

**Figure 6:**
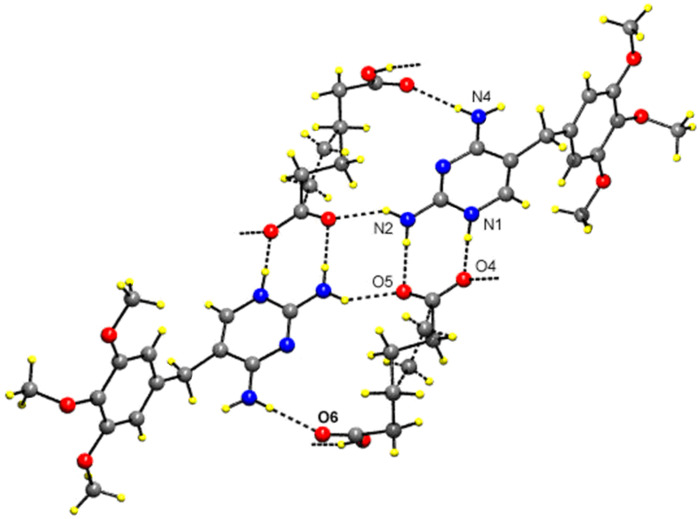
The hydrogen-bonded DDAA array in the compound **2**.

**Figure 7:**
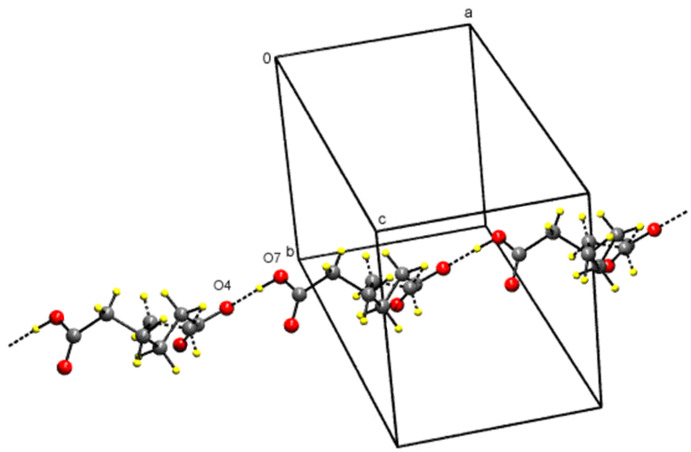
The supramolecular chain made up of hydrogen adipate in the compound **2**.

In the compound **2** (Table 1), in motif V, two TMP cations and two hydrogen adipate anions are arranged about an inversion center so that the complementary DDAA arrays of quadruple hydrogen-bonding patterns are formed. This has also been observed in TMP m-chlorobenzoate [11], TMP-hydrogen glutarate [17] and TMP succinate [29]. In motif VI, the hydrogen atoms of 2- and 4-amino groups of the TMP cation are hydrogen-bonded to the carboxylate and carboxyl ends, respectively, of the same hydrogen adipate ion. Thus, the hydrogen adipate bridges the 2-amino and 4-amino groups of TMP. These hydrogen-bonded interactions are almost identical with TMP-dicarboxylate salts such as TMP-hydrogen glutarate [17] and TMP-succinate [29] but differ only in the number of carbon atoms of the chain. Such cyclic hydrogen-bonded ring formation blocks the base-pairing interaction between the pyrimidine moieties. Hence base-pairing has not been observed in the crystal structures of trimethoprim-hydrogen glutarate [17] and TMP-succinate [29] and compound **2**. The supramolecular sheet structure for this compound **2** is shown in Figure 6. The carboxyl group (O7-H) of the hydrogen adipate is hydrogen-bonded to the carboxylate group (O4) of the neighbouring hydrogen adipate ion(motif VII). This head-to-tail arrangement (carboxyl-carboxylate interaction) of the hydrogen adipate ions leads to hydrogen-bonded supramolecular chain. This is shown in Figure 7.

The internal angles at N1 (C2-N1-C6) in the protonated pyrimidine ring of the compounds **1** and **2** are 119.9(1)° and 119.6(2)° respectively, the corresponding angle in the neutral trimethoprim(TMP) molecule [31] being 115.5°. Such an enhancement of internal angle at the site of protonation of pyrimidine ring is very characteristic. In the compounds **1** and **2** the dihedral angles between the plane of the pyrimidine and phenyl rings are 74.0(7)° and 88.8° respectively. These values are closer to the crystal structures of TMP-sulfate trihydrate [20] (75.8(9)°) and TMP 4-hydroxybenzoate dihydrate [30] (89.1(1)°).

The major (77%) and minor (23%) components in the disordered hydrogen adipate molecule adopt quite unusual bent carbon chain conformations: the *gauche-gauche-trans (ggt)* and the *gauche-trans-trans (gtt)* forms, respectively. Of the 46 adipic acid fragments present in the Cambridge Crystallographic Data Base [32] there is only one example of the *ggt* conformation [33] and two cases in which the acid adopts the *gtt* form [34–35]. The adoption of the bent carbon chain conformation by adipic acid seems necessary in order to place the two terminal carboxyl functions in mutual *syn* orientation so that they can fasten the 2- and 4-amino groups of the TMP molecule. The disorder, on the other hand, might result from incompatible dimensions between the adipic acid and the two amino groups of the TMP molecule. Much better fit between the 2- and 4-amino groups of the TMP molecule on one side, and aliphatic dicarboxylic acid on the other side is achieved in the case of glutaric acid [17]. This is for two reasons: firstly, in the energetically preferred extended carbon chain conformation an odd number of carbon atoms in a chain implicates the syn orientation of the terminal carboxyl functions and, secondly, the carbon chain is identical in length as the N2-C2-N3-C4-N4 fragment of the TMP. The observation that, irrespective on the number of carbon atoms constituting the dicarboxylic acid chain, the TMP/dicarboxylic acid interactions are represented by the same motif VI is quite unusual.

**Figure 8:**
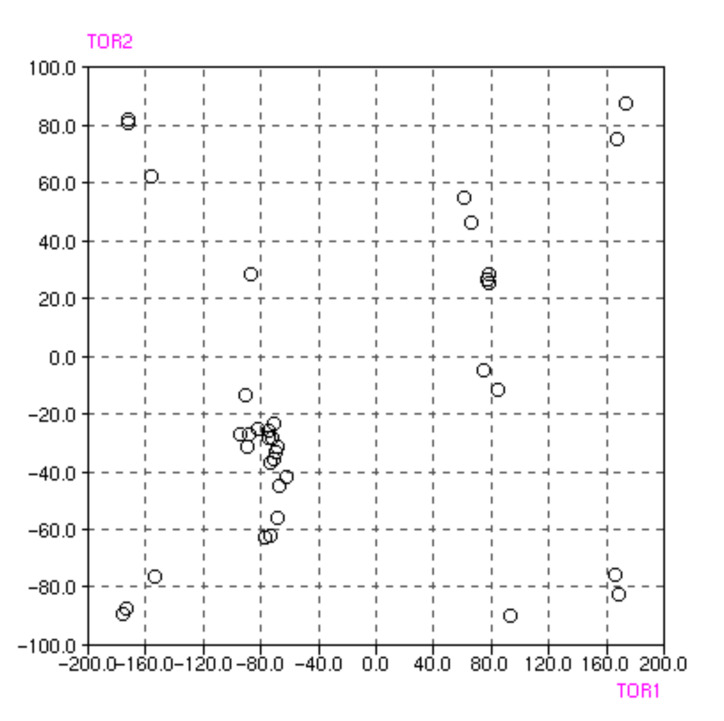
Scatterplot illustrating the distribution of the two torsion angles (C4-C5-C7-C8 (TOR1) and C5-C7-C8-C9 (TOR2)) that describe the mutual orientation of pyrimidine and phenyl rings in the TMP molecule. The torsion angle values were obtained from the July 2003 release of the 5.24 version of the CSD [32]. The values of TOR2 have been restricted to the range from -90 to +90°.

The TMP molecule can be regarded as having a rigid frame, built on the methyl group, on which the substituted phenyl and pyrimidine six-membered rings are free to rotate. An arbitrary conformation of this molecule can be described by the torsion angles of the two rings to the frame. We define these torsion angles as C4-C5-C7-C8 and C5-C7-C8-C9, i.e. with respect to one of the rings the other can rotate around the C5-C7 or around the C7-C8. Figure 8 shows the distribution of these torsion angles in 37 TMP fragments deposited in the Cambridge Structural Data Base [32]. The points mostly cluster around the plus/minus (80°, 30°) and (160°, 70°) regions. The points representing the (80°, 30°) combination predominantly lie in the region where both torsion angles have the same sign, which is the condition for a propeller conformation. In the presented crystal structures **1** and **2** the corresponding torsion angles adopt the values -161.4(1) and 63.5°, and 69.1(2) and 36.5(3)°, respectively. Hence the observed TMP conformations match the two most densely populated conformations observed in other crystal structures containing the TMP moieties.

In the compound **1**, the ionized and non-ionized carboxyl groups are inclined at an angles of 6.0(1)° and 9.0(1)° respectively to the plane of the phenyl ring. The bond angles of C19-C17-O4, C19-C17-O5 in the carboxyl group are 119.1(2)° and 120.7(17)° respectively. The similar angles at the carboxylate group C20-C18-O6 and C20-C18-O7 are 120.4(2)° and 117.4(2)° respectively. These values are comparable with the crystal structure of pyrimethamine hydrogen phthalate [21]. In the compound **2**, the bond angles at carboxylate group, C18-C17-O5 and C18-C17-O4 are 120.1(2)° and 116.5(2)° respectively, whereas the angle at the carboxyl group C21-C22-O6, C21-C22-O7 are 123.1(2)° and 113.1(2)° respectively.

The crystal structure of compound **2** is further stabilized by two C-H...π interactions [38] [C16-H161...Cg1(atoms N1-C6) (2.923Å, 137°) and C20-H201...Cg2 (atoms C8-C13) (2.779Å, 156°)] and the pyrimidine stacking interactions. The interplanar and centroid to centroid distances are 3.381Å and 3.738Å, respectively, and the angle between the centroid vector and normal to the plane is 25.3°.

## Supporting Information

File 1 the CIF information

File 2 experimental details
