# Supplementary material for: Crystal engineering of analogous and homologous organic compounds: hydrogen bonding patterns in trimethoprim hydrogen phthalate and trimethoprim hydrogen adipate
Source: Beilstein J Org Chem. 2006 Apr 7;2:8. doi: 10.1186/1860-5397-2-8 (PMC1470456; doi:10.1186/1860-5397-2-8)
Supplement: File 2 — experimental details [file Beilstein_J_Org_Chem-02-08-s002.doc]

**Experimental:**

The compound 1: Hot aqueous solution of phthalic acid (80mg, LOBA) was mixed with hot n-propanol-water (75%/25%) solution of trimethoprim(TMP) (145mg, Shilpa Antibiotics Ltd) in 1:1 molar ratio. The solution was warmed over a water bath for an hour. On cooling, the material was obtained as a precipitate. The colourless plate-like crystals were obtained by recrystallization from acetone-ethanol (50%/50%) mixture. Compound 2 : Hot methanolic solutions of TMP (72mg, Shilpa Antibiotics Ltd) and adipic acid (37mg, LOBA) were mixed in 1:1 molar ratio and warmed over a water bath for half an hour. The colourless plate-shaped crystals were obtained by slow evaporation.

**X-ray crystallography**: The X-ray data were collected for the compounds 1 and 2 at 293°K on a Kuma KM-4 four circle diffractometer[23] by using a graphite monochromated CuKα radiation. All the non-hydrogen atoms were refined anisotropically. All the hydrogen atoms were located from difference Fourier map and refined isotropicaly. There are two disordered carbon atoms C18a and C19a (In compound 2) which were assigned partial occupancies. The respective hydrogen atoms of these carbons were treated as riding atoms. The crystal data and details of structural determination for the compounds 1 and 2 are listed in table 1. CCDC reference numbers : 209281 and 221848
